# Breaking Barriers in the Management of Amniotic Fluid Embolism With Interventional Radiology: A Case Report

**DOI:** 10.7759/cureus.67821

**Published:** 2024-08-26

**Authors:** Amber W Sun, Priya Barua, Alexander Benton, Brian Do

**Affiliations:** 1 Department of Radiology, University of Missouri - Kansas City (UMKC) School of Medicine, Kansas City, USA; 2 Department of Obstetrics and Gynecology, University of Missouri - Kansas City (UMKC) School of Medicine, Kansas City, USA; 3 Department of Interventional Radiology, University Health Truman Medical Center, Kansas City, USA

**Keywords:** ir, afe, anaphylactoid syndrome of pregnancy, interventional radiology management, amniotic fluid embolism

## Abstract

Amniotic fluid embolism (AFE) is a rare obstetric emergency with a high mortality rate despite treatment. The pathogenesis likely involves inflammatory cytokines reacting to amniotic fluid in the bloodstream, causing rapid multi-organ failure and coagulopathy. Prompt recognition and supportive, multidisciplinary treatment are vital for enhancing patient outcomes. This report presents the case of a 27-year-old female with acute decompensation due to AFE. Our patient was scheduled for cesarean delivery due to high-risk placental anomalies and began demonstrating signs of AFE and severe hemorrhage shortly after delivery. She was transferred to our tertiary care center and was treated with coil embolization of the hemorrhaging uterine, iliac, and epigastric arteries, ultimately stabilizing her and saving her life. This case highlights the successful management of AFE with interventional radiological (IR) techniques.

## Introduction

Amniotic fluid embolism (AFE) is a rare medical emergency that can occur before labor, during labor, and postpartum. The pathogenesis is theorized to be due to the release of inflammatory cytokines, such as histamine, endothelin, and leukotrienes, reacting to amniotic fluid debris in the maternal bloodstream. This debris can include squamous cells, trophoblastic cells, mucin, and lanugo [1,2]. Abnormal placentation, such as placenta previa or placenta accreta, increases the risk of an inflammatory response and facilitates large volumes of amniotic fluid entering the maternal bloodstream [3]. In a study of women diagnosed with AFE, 10% of these patients had an obstetric history complicated by placenta previa [3].

In the pulmonary vasculature, the widespread inflammatory response triggers both pulmonary vasoconstriction and bronchoconstriction, resulting in acute obstruction of the pulmonary arteries and pulmonary edema. Consequently, the right heart chambers enlarge in response to the blockage in the pulmonary arteries, precipitating tricuspid valve insufficiency and subsequent acute right-sided heart failure [4]. The left ventricle also experiences significant functional impairment due to myocardial ischemia from acute right-sided heart failure, leading to acute systolic dysfunction, reduced cardiac output, hypoxemia, and hypotension [5,6]. 

Amniotic fluid also activates coagulation and fibrinolytic factors, potentially triggering disseminated intravascular coagulation (DIC) in approximately 80% of cases. The presentation of coagulopathy varies, with onset ranging from immediately after cardiovascular collapse to later in the course [6]. DIC manifests as bleeding from venipuncture or surgical sites, hematuria, GI hemorrhage, or vaginal bleeding [7].

Amniotic fluid debris can induce uterine atony by infiltrating the myometrium and triggering local inflammation. An alternative theory suggests that debris entering via uterine incisions may travel through the uterine arteries to the internal iliac artery, and onward to the common iliac artery, exacerbating hemorrhage by causing both uterine atony and pelvic arterial vasculature extravasation [8,9].

The prognosis of AFE is grim. Studies suggest that maternal mortality ranges from 20% to 60% [10]. In patients with placenta accreta spectrum disorder, mortality rates can double [11]. In the United States, AFE is the second leading cause of peripartum maternal death and affects 2.2 to 7.7 per 100,000 deliveries [12].

Diagnosis is based on the clinical triad of hypoxia, hypotension, and coagulopathy in peripartum or postpartum women [13]. Beyond vital signs, the initial evaluation of AFE includes performing transesophageal echocardiography to assess cardiopulmonary collapse. Labs, including a complete blood count and coagulation panel, are essential to determine the presence of DIC [14].

The Society for Maternal-Fetal Medicine and the Amniotic Fluid Embolism Foundation have attempted to establish uniform diagnostic criteria for AFE research. These criteria include the sudden onset of cardiorespiratory arrest or hypotension (systolic blood pressure <90 mmHg) with respiratory compromise (dyspnea, cyanosis, or SpO_2_ <90%), followed by the presence of DIC using the scoring system developed by the International Society on Thrombosis and Haemostasis, modified for pregnancy. Coagulopathy must occur before significant blood loss can account for consumptive coagulopathy. The clinical onset typically occurs during labor or within 30 minutes of placental delivery, and the patient should not have a fever (≥38.0°C) during labor [15].

Treatment is supportive, including respiratory, cardiac, hemostatic, and hemodynamic resuscitation. Regarding obstetrical management, for antepartum patients over 23 weeks, urgent cesarean delivery may be indicated. This requires a multidisciplinary team to be heavily involved in the patient’s care, with primary contributions from obstetrics, critical care, cardiology, and pulmonology [16].

Optimal management of acute onset heart failure includes agents that target cardiac preload, hypotension, and contractility. Commonly administered inotropic medications include dobutamine or milrinone [16]. Inotropes, along with sildenafil and prostacyclins, also reduce pulmonary vascular resistance and consequently decrease right ventricular afterload. Hypotension can be managed with vasopressors, such as norepinephrine or vasopressin. In the setting of volume overload, fluids should be avoided to prevent over-distention of the ventricles. Noninvasive mechanical ventilation or intubation should be considered to manage cardiogenic pulmonary edema. Diuretics may also be indicated [7].

Management of severe hemorrhage and DIC can require aggressive resuscitation with packed red blood cells (pRBCs), fresh-frozen plasma (FFP), and platelets, administered at a 1:1:1 ratio. Tranexamic acid can also be used for fibrinolysis [17].

This case report describes a case of AFE successfully treated with interventional radiology (IR) coil embolization. In July 2024, a literature review of the PubMed database using the terms “amniotic fluid embolism,” “AFE,” “interventional radiology,” and “IR” in various combinations produced no results, indicating that, to the best of our knowledge, this may be the first reported case of AFE treated using IR techniques.

This article was previously presented as a poster at the 2024 University of Missouri - Kansas City Health Sciences Student Research Summit, on March 20, 2024.

## Case presentation

The patient is a 27-year-old pregnant G2P1 female who initially presented at an affiliated hospital with vaginal bleeding at 34 weeks gestation, attributed to a previously diagnosed placenta previa. The pregnancy was otherwise complicated by Rh negativity, which was treated with Rh immune globulin. The patient has a past medical history of an unmedicated and unspecified seizure disorder, unmedicated major depressive disorder, pyelonephritis, mild intermittent asthma, and unspecified anemia. At that time, she was admitted for observation. After discharge, during this admission, the care team planned for a low transverse cesarean section with the high-risk obstetrics team at 36 weeks gestation.

During delivery, the patient developed severe bradycardia, with a heart rate in the range of 30 beats per minute, which improved with glycopyrrolate (0.2 mg) and atropine (0.4 mg). Shortly after this intervention, she developed tachycardia, with a heart rate in the range of 130 beats per minute. The patient denied shortness of breath, and her oxygen saturation and blood pressure remained stable. Approximately 10 minutes later, she experienced an episode of pulseless ventricular tachycardia lasting 30 seconds that resolved without intervention. Otherwise, the cesarean section was completed without significant complications, with an estimated blood loss (EBL) of 700 mL. 

In the recovery room, the patient began experiencing increased vaginal bleeding. Fundal massage, oxytocin, misoprostol, methylergonovine, and carboprost were administered without controlling the bleeding. Attempts to place a JADA device (Organon, Jersey City, NJ, USA) were repeatedly unsuccessful due to insufficient dilation of the cervix. A Cook catheter (Cook, Bloomington, IN, USA) was inflated with 80 mL of normal saline to slow the bleeding. The patient continued to deteriorate rapidly, with blood pressures becoming hypotensive in the 60-90/40-60 mmHg range and tachycardia in the range of 110-130 beats per minute. Oxygen saturation remained above 95% without supplemental oxygen. Her mental status began to deteriorate, and she experienced brief seizure-like activity. Point-of-care abdominal ultrasound demonstrated a uterine clot and free fluid in the bilateral upper quadrants. A rapid echocardiogram showed a reduced ejection fraction of 35-40% and a troponin level of 50,738.7 pg/mL.

At this time, the decision was made to transfer the patient to our tertiary care center. During transportation, blood pressures ranged from 90/50 mmHg, and oxygen saturation decreased from 100% to 70%, prompting the administration of 5 L of oxygen supplementation through a nasal cannula (NC). On arrival, her clinical state remained poor. Vitals on arrival were significant for similar blood pressures and heart rate, with 100% oxygen saturation on 5 L of supplemental oxygen via the NC. She never lost mentation and was conversational during transport and admission. A complete blood panel, coagulation tests, and fibrinogen levels revealed a hemoglobin level of 7.6 g/dL (compared to a pre-operative level of 9.6 g/dL), platelets of 146,000 per μL, fibrinogen of 69 mg/dL, and an INR of 1.2, consistent with DIC and suspicious for AFE. Focused Assessment with Ultrasonography in Trauma (FAST) showed diffuse hemoperitoneum, and a chest X-ray (CXR) demonstrated pulmonary edema. Rotational thromboelastometry (ROTEM) showed severe coagulopathy. In the setting of large volume loss and coagulopathy, transfusion of cryoprecipitate, platelets, pRBCs, and FFP was performed. Volume resuscitation was supplemented with phenylephrine and norepinephrine.

**Figure 1:**
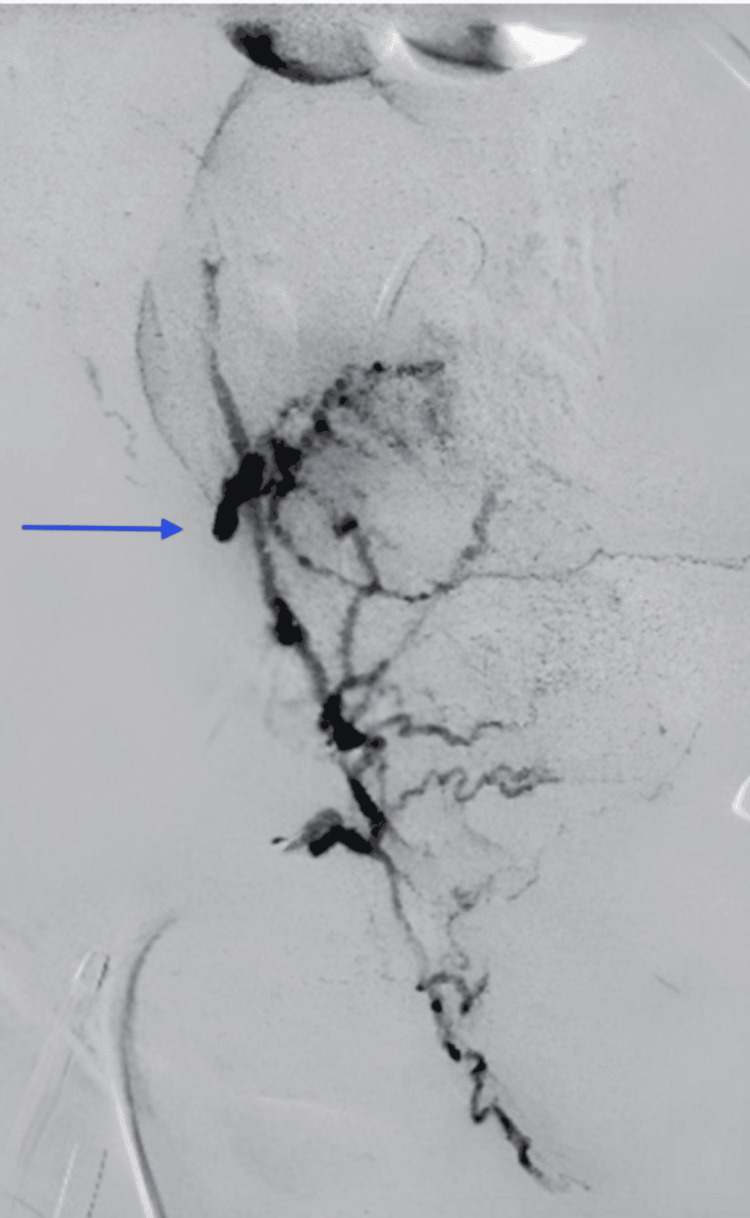
Digital subtraction angiogram of the pelvis reveals a globular collection of contrast, which pooled on delayed images (not shown), consistent with active arterial extravasation of the right uterine artery.

Initially, plans were made to undergo computed tomography (CT) angiography of the chest, abdomen, and pelvis to exclude a pulmonary embolism and identify a possible hemorrhagic source. However, due to severe hemodynamic instability during the initial stages of the study, she was taken to the IR suite for embolization of the bilateral uterine and internal iliac arteries. The decision to forgo anesthesia support was made collectively by all medical teams involved, as the risks of delaying care to await anesthesia involvement would have outweighed any benefit. A digital subtraction angiogram of the pelvis revealed abnormal pooling of contrast involving the right uterine artery, consistent with active arterial extravasation (Figure 1). Particle and coil embolization of the right and left uterine arteries was performed, followed by empirical particle embolization of the bilateral internal iliac arteries. A post-procedure digital subtraction angiogram was obtained to confirm successful hemostasis.

Due to ongoing hemorrhage attributed to the AFE throughout the case, the patient underwent volume resuscitation with pRBCs, FFP, and platelets in a 1:1:1 fashion, with cryoprecipitate administered post-procedurally due to abnormal ROTEM results. After volume resuscitation and embolization, she did not require vasopressor support for her vital signs to normalize in the immediate post-procedure period.

Later that evening, she became spontaneously hypotensive and tachycardic, with reports of severe abdominal pain and chest pain, requiring extensive blood products to maintain a mean arterial pressure (MAP) >60 mmHg. Bedside CXR demonstrated progression of bilateral pulmonary opacities in the setting of oxygen desaturation and dyspnea. She underwent emergency endotracheal intubation and was given furosemide, three units of pRBCs, three units of FFP, and one unit of cryoprecipitate.

**Figure 2:**
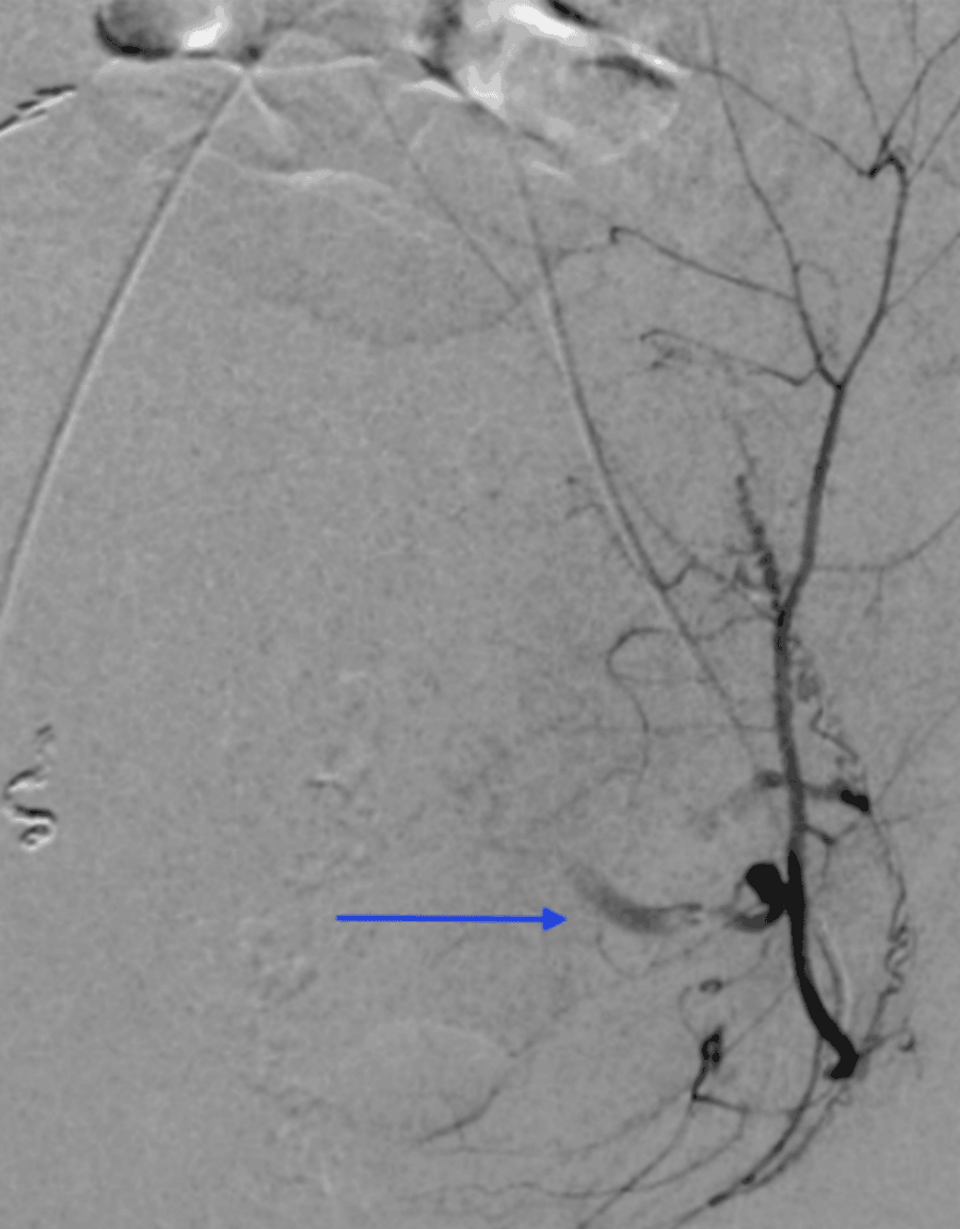
Second-look digital subtraction angiogram of the pelvis reveals active extravasation of the left inferior epigastric artery.

**Figure 3:**
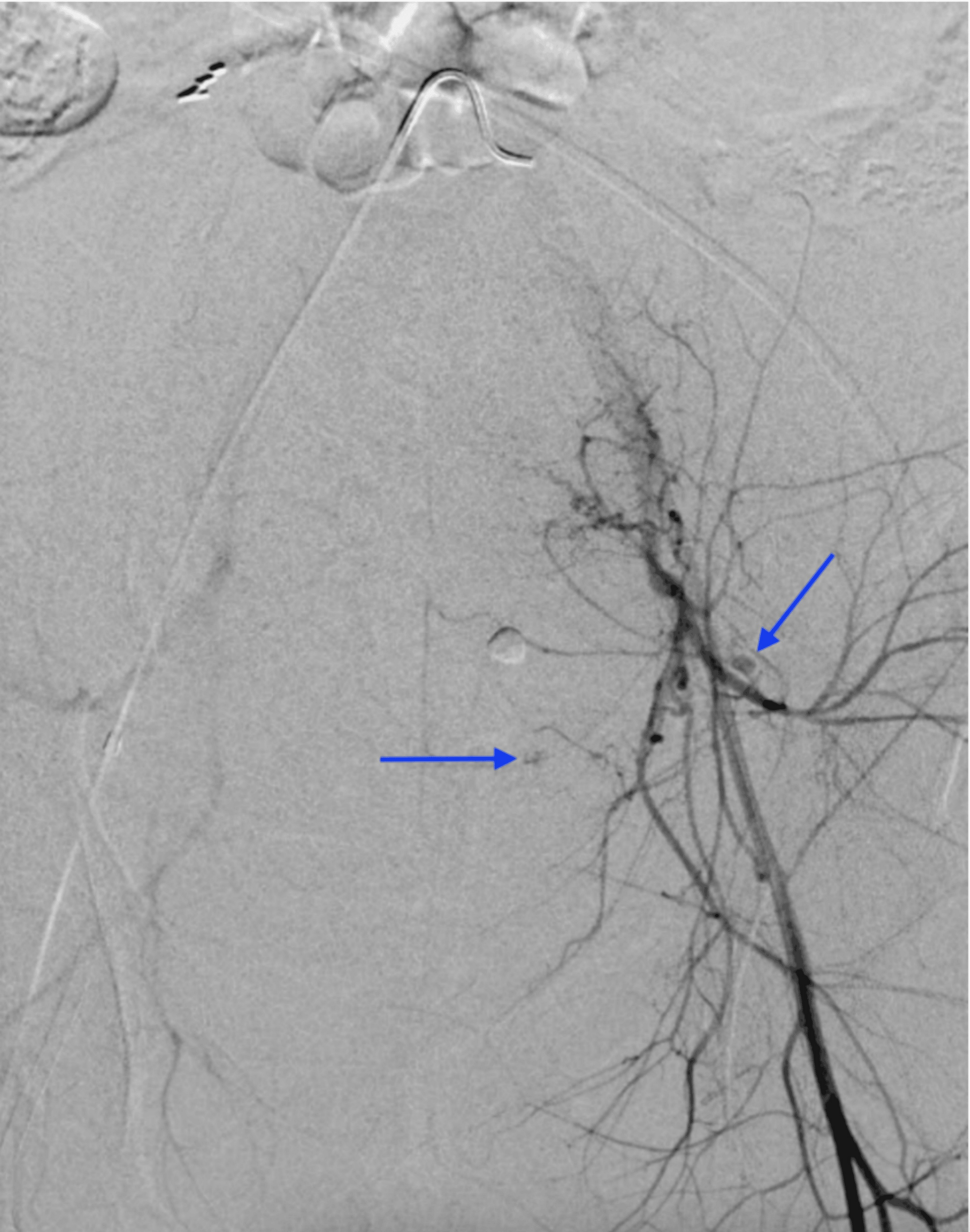
First digital subtraction angiogram reveals subtle extravasation of the inferior epigastric artery, which was overlooked on initial patient presentation.

**Figure 4:**
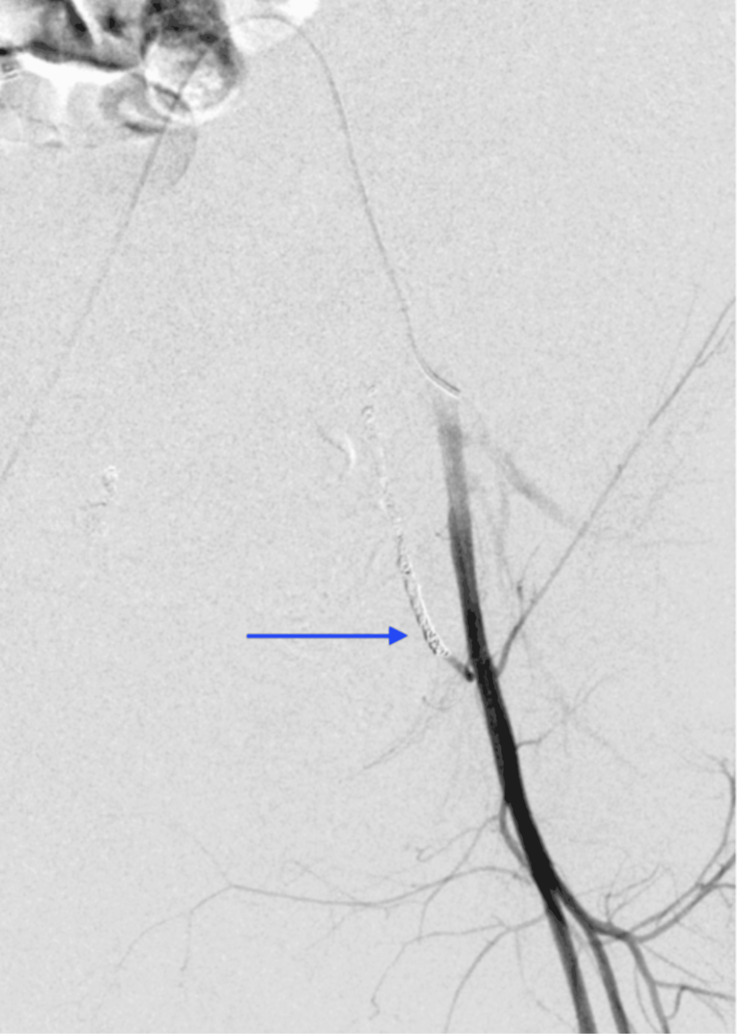
Second-look digital subtraction angiogram of the pelvis shows coil embolization of the left inferior epigastric artery.

After her hemodynamic status improved, a multidisciplinary discussion was held between the obstetrics, pulmonology, cardiology, and IR teams. The decision was made to take the patient for a second-look pelvic angiogram over CT angiography since angiography can be diagnostic and potentially therapeutic. Similar to the first IR procedure, the decision to forgo anesthesia support was made collectively by all teams involved, as the risks of delaying care would have outweighed any benefit. The second-look angiogram revealed brisk arterial extravasation from the left inferior epigastric artery (Figure 2), which was inconspicuous on the initial angiogram (Figure 3). IR then performed coil embolization of the left inferior epigastric artery (Figure 4) and repeated the empiric particle embolization of the left and right internal iliac arteries. A conventional pulmonary arteriogram was also obtained to exclude a large pulmonary embolus based on her worsening hypoxemia. This study was negative for large filling defects and demonstrated adequate pulmonary perfusion. The post-embolization angiogram revealed stasis of flow in the left inferior epigastric artery and bilateral internal iliac arteries.

She was subsequently stabilized and remained so for the remainder of her admission. For volume resuscitation during her admission, she received in total: 16 units of pRCBs, 13 units of FFP, 2 units of platelets, and 4 units of cryoprecipitate. The cardiology team discharged her with losartan 25 mg, carvedilol 3.125 mg, and enoxaparin 40 mg daily for six weeks.

Since discharge, she has been regularly following up with outpatient cardiology. Shortly after discharge, her ejection fraction improved to 45-50%, with residual akinesis of the apical septum and anterior myocardial segments. She was put on a Zio patch (iRhythm, San Francisco, CA, USA) for two weeks to monitor for arrhythmia. The monitor revealed an average sinus rhythm of 77 beats per minute and two episodes of nonsustained ventricular tachycardia (NSVT), lasting five beats each. Three months after discharge, her ejection fraction showed continued improvement to an estimated 60%. The patient continues to take carvedilol 3.125 mg daily.

## Discussion

IR’s prompt identification and treatment of arterial extravasation were critical for stabilizing this patient and mitigating the risk of further clinical deterioration. In obstetric emergencies, especially those involving severe bleeding, time is of the essence, and precision is mandatory. IR potentially offers minimally invasive techniques that can effectively and quickly control emergent bleeding and provide patient stabilization without the need for vasopressor support or general anesthesia. The benefits of IR in such scenarios are manifold: reduced recovery times, decreased risk of infection, and potential reduction of morbidity compared to traditional surgical approaches. Opting for an IR procedure rather than an exploratory laparotomy is beneficial, as it is less traumatic and minimizes stress on the patient. IR procedures can be more time-efficient and involve less extensive preparation compared with traditional surgical preparation. In the IR suite, specialized staff can quickly don lead protection and provide precise patient care with a streamlined workflow. In cases where vascular territory allows and there’s no evidence of extravasation, IR can quickly identify the responsible artery but may face challenges navigating the catheter due to anatomical variants; in such situations, empirical embolization is often performed since the pelvic vasculature can be generally forgiving.

Embolic agents can be broadly classified into two categories: temporary or permanent agents. Temporary agents are resorbable and include gelatin sponge and microfibrillar collagen particles. Permanent agents include platinum coils and polyvinyl alcohol particles. The decision to use microfibrillar collagen particles was due to the ability to embolize a large vascular distribution at the arteriolar/capillary level. This was advantageous when embolizing the internal iliac artery and its branches. The decision to use platinum coils for the left inferior epigastric artery embolization was primarily for precise, predictable placement.

There is known versatility of IR procedures in the field of obstetrics, such as their application in postpartum hemorrhage. This cross-applicability suggests that training and resource allocation towards IR could benefit a wider range of obstetric emergencies than previously known, enhancing overall maternal care.

Currently, no other research or case documentation highlights the potential significance of IR procedures in managing AFE. Further studies are warranted to analyze the impact of incorporating IR into the standard management of AFE across medical facilities and to validate the efficacy of IR in this context. There should also be an investigation into whether IR offers advantages over traditional open surgery. Specifically, it should assess IR's potential to enhance patient outcomes, alleviate the burden on critical care resources, and streamline the overall management of AFE. Additionally, future research should focus on comparative studies that evaluate patient outcomes with and without the use of IR in AFE cases, providing a robust evidence base for its incorporation into clinical guidelines.

## Conclusions

While IR may not traditionally be a primary component of the management for AFE, its inclusion in this patient’s care optimized their outcome in an obstetric emergency with a typically poor prognosis. The prompt integration of IR into a multidisciplinary approach to managing the obstetric emergency of AFE played a pivotal role in the survival of this patient, highlighting the significant impact IR can have on optimizing patient outcomes.
